# The prevalence of CT-defined low skeletal muscle mass in patients with metastatic cancer: a cross-sectional multicenter French study (the SCAN study)

**DOI:** 10.1007/s00520-021-06603-0

**Published:** 2021-12-03

**Authors:** Bruno Raynard, Frederic Pigneur, Mario Di Palma, Elise Deluche, François Goldwasser

**Affiliations:** 1 Gustave-Roussy, 114 Rue Edouard Vaillant, 94800 Villejuif, France; 2 Henri Mondor University Hospitals, AP-HP Créteil, France; 3 American Hospital of Paris, Neuilly-sur-Seine, France; 4 Limoges University Hospital, Limoges, France; 5 Cochin Hospital, AP-HP, CARPEM, Paris Descartes University, Paris, France

**Keywords:** Cachexia, Low muscle mass, Skeletal mass index (SMI), Nutritional support

## Abstract

**Background:**

Cachexia, characterized by involuntary muscle mass loss, negatively impacts survival outcomes, treatment tolerability, and functionality in cancer patients. However, there is a limited appreciation of the true prevalence of low muscle mass due to inconsistent diagnostic methods and limited oncologist awareness.

**Methods:**

Twenty-nine French healthcare establishments participated in this cross-sectional study, recruiting patients with those metastatic cancers most frequently encountered in routine practice (colon, breast, kidney, lung, prostate). The primary outcome was low skeletal muscle mass prevalence, as diagnosed by estimating the skeletal mass index (SMI) in the middle of the third-lumbar vertebrae (L3) level via computed tomography (CT). Other objectives included an evaluation of nutritional management, physical activity, and toxicities related to ongoing treatment.

**Results:**

Seven hundred sixty-six patients (49.9% males) were enrolled with a mean age of 65.0 years*.* Low muscle mass prevalence was 69.1%. Only one-third of patients with low skeletal muscle mass were receiving nutritional counselling and only 28.4% were under nutritional management (oral supplements, enteral or parenteral nutrition). Physicians highly underdiagnosed those patients identified with low skeletal muscle mass, as defined by the primary objective, by 74.3% and 44.9% in obese and non-obese patients, respectively. Multivariate analyses revealed a lower risk of low skeletal muscle mass for females (OR: 0.22, *P* < *0.01*) and those without brain metastasis (OR: 0.34, *P* < *0.01*). Low skeletal muscle mass patients were more likely to have delayed treatment administration due to toxicity (11.9% versus 6.8%, *P* = *0.04*).

**Conclusions:**

There is a critical need to raise awareness of low skeletal muscle mass diagnosis among oncologists, and for improvements in nutritional management and physical therapies of cancer patients to curb potential cachexia. This calls for cross-disciplinary collaborations among oncologists, nutritionists, physiotherapists, and radiologists.

**Supplementary Information:**

The online version contains supplementary material available at 10.1007/s00520-021-06603-0.

## Introduction

Cancer cachexia is a complex metabolic syndrome characterized by involuntary muscle loss with or without loss of fat mass, systemic inflammation, and negative protein and energy balance. This condition cannot be completely reversed with conventional nutritional care and leads to progressive functional impairment in cancer patients [1–3]. Cancer cachexia is associated with an increased risk of postoperative complications, increased mortality in the metastatic phase, increased treatment intolerance, longer hospitalizations, and alterations in patient quality of life [4–7]. Deleterious effects increase with severity, delaying cancer management. Early care, prior to 5% bodyweight loss, is thus recommended [7].

The most clinically relevant phenotype of cachexia is sarcopenia. In oncology, the diagnosis of sarcopenia is generally based solely upon the loss of skeletal muscle mass. Dual-energy x-ray absorptiometry (DEXA) is the method of reference for measuring muscle mass [8–10]. More recently, estimations of the skeletal mass index (SMI) at the third-lumbar vertebra (L3) level via computed tomography (CT) have been used to define low muscular mass [11–13]. Although this particular application of CT is not yet routinely implemented in France, it has been proposed as an objective measure for the identification of low skeletal muscle mass among cancer patients [14].

Methods of cachexia diagnosis are variable; Fearon et al. [3] propose three different diagnostic criteria, one of which is defined by CT-defined low muscle mass: (1) > 5% weight loss in the last 6 months, (2) BMI < 20 kg/m^2^ and ongoing weight loss > 2%, or (3) sarcopenia, in particular as defined by low SMI (measured by CT segmentations at the midpoint of the L3 vertebrae, using the thresholds of < 55cm^2^/m^2^ in males and < 39 cm^2^/m^2^ in females [3]) and ongoing weight loss > 2%. These SMI thresholds have since been endorsed by the European Society for Clinical Nutrition and Metabolism [15].

Precise estimates of low muscle mass prevalence among cancer patients remain scarce and vary widely. As such, the primary objective of this cross-sectional, multi-centric study conducted in France was to evaluate the prevalence of low skeletal muscle mass as measured by L3 cross-sectional CT scans in patients diagnosed with metastatic cancers. Moreover, misdiagnoses by oncologists and nutritional strategies available to cancer patients were explored.

## Methods

### Study population and design

This study was conducted in accordance with the Declaration of Helsinki. All persons provided their informed consent prior to enrollment. The protocol was in line with French data protection regulations (CNIL, approval no. 2066086) and approved by the French ethical research committee, “Le Comité de Protection des Personnes” (CPP), on the 6 July 2017 (approval no. 2017-A01648-45).

This cross-sectional study was conducted within the investigators’ oncology practices in 29 public and private hospitals in France, between September and October 2017. Patients were males or females aged ≥ 18 years, diagnosed with metastatic lung, kidney, colon, breast, or prostate cancer, irrespective of the age of the diagnosis. At the time of inclusion, patients had to be undergoing chemotherapy, targeted therapy, hormonotherapy, or immunotherapy that was initiated since at least one cycle of treatment or for at least 1 month, irrespective of the line of ongoing treatment. Patients had to have a CT scan including an L3 cross-section suitable for SMI evaluation of low muscle mass, performed (for any reason) between 6 weeks before and 4 weeks after inclusion to the study. Patients were excluded if diagnosed with two or more malignant pathologies, if being treated exclusively by radiotherapy, if they had surgery in the previous 30 days, or if they had a concomitant neurological comorbidity, other than cerebral metastasis.

The investigating pair in each of the 29 centers consisted of an oncologist-radiologist duo. An obligatory training presentation was given to investigating radiologists for the measurement of the cross-sectional skeletal muscle area (CSA) using CT. This training was provided by a radiologist member of the SCAN study scientific committee using a standardized approach, in order to minimize the risk of observer error in CSA slice selection and in contouring the borders of the skeletal muscles [16]. Muscle mass was determined using manual segmentation on dedicated post-treatment stations (FujiFilm [Synapse 3D], Siemens [*syngo*.via version VB10 and VB20], GE Healthcare or Toshiba Medical) as a CSA in the middle of L3 level [17]. It should be noted that the decision to permit the use of different post-treatment stations is supported by data from a study that compared 4 different imagery station software and demonstrated an excellent correlation between readings, with limited inter- and intra-operator variability [18]. The total CSA was measured in square centimeters with a pre-established density threshold within − 29 to + 150 Hounsfield units (HU; including the external and internal obliques, paraspinal, rectus abdominis, transversus abdominis, and psoas muscles). All CSA were individually normalized for stature, as is conventional for body composition evaluation, resulting in the SMI: SMI = CSA/height^2^ (cm^2^/m^2^) [16, 19]. These SMI were subsequently evaluated by the investigating radiologist.

Oncologists, who were blinded to the CT scan results, recorded the following information during the inclusion visit: current weight, weight 1 and 6 months ago, height, dietary and nutritional support for each patient, weekly physical activity, food intake assessment at the previous meal using a visual analogue scale (VAS, wherein 10 signifies normal, healthy food intake for the patient) [20], and performance status (PS) scores using the Eastern Cooperative Oncology Group (ECOG) scale of 0 (fully active) to 5 (death) [21]. C-reactive protein (CRP) and serum albumin (SA) data were collected from test results between a month before and a week after the CT scan to assess malnutrition in line with local guidelines [22] and the patients’ Glasgow prognostic scores (GPS), categorizing patient prognosis on a scale of 0 (best) to 2 (worst) [23].

Malnutrition severity was defined according to criteria that were in use in routine practice. These criteria were defined by the French national health authorities [22], using the thresholds listed below by age group: moderate malnutrition < 70 years old, BMI ≤ 18.5 to 16 kg/m^2^ or SA < 30 to 20 g/L, and ≥ 70 years old, BMI < 21 to 18 kg/m^2^ or SA < 35 to 30 g/L; and severe malnutrition < 70 years old, BMI ≤ 16 kg/m^2^ or SA < 20 g/L, and ≥ 70 years old, BMI < 18 kg/m^2^ or SA < 30 g/L.

Additionally, the oncologist was required to assess for low skeletal muscle mass as per their discretion (no predefined method), while being blinded to the CT results.

### Study objectives

The primary objective was to determine the prevalence of low skeletal muscle mass in patients with metastatic cancer of the lung, kidney, colon, breast, or prostate, by estimating on CT the skeletal muscle index (SMI) in the middle of the L3 level, with cut-off values < 55cm^2^/m^2^ and < 39 cm^2^/m^2^ indicating low muscle mass in males and females, respectively [3].

Other outcomes included (1) an evaluation of the level of agreement between CT measurements and oncologists’ evaluations of low muscle mass (for the total study population, and for obese (body mass index [BMI] ≥ 30 kg/m^2^) and non-obese sub-groups (BMI < 30 kg/m^2^); (2) a description of nutritional care in cancer patients (proportion of patients receiving nutritional counselling and nutritional management); (3) weekly physical activity of cancer patients (equivalent to 30 min of walking or cycling on flat terrain, or swimming), and daily hours spent in bed as reported by the patient; and (4) an evaluation of toxicities related to ongoing anti-cancer treatment and their impact on dosage modifications, treatment delays due to toxicity, treatment interruptions, and the occurrence of adverse events (AE) ≥ grade 3. AEs were graded according to the Common Terminology Criteria for Adverse Events (CTCAE).

Cachexia prevalence in patients with low muscle mass was estimated in an exploratory manner according to each of the three criteria defined by Fearon et al. [3], which were elaborated in the introduction. Multivariate analyses of the relationship between the presence of low muscle mass and patient age, sex, primary tumor site, weight loss, current BMI, serum albumin, PS score, VAS for food intake, and brain metastasis were also performed.

### Statistical analyses

Data management and statistical analyses were performed by Kantar Health (Paris, France), using DAISIE (version 2.4.25 & 2.4.45) and R i386 3.0.1. Descriptive analyses (means, SD, median, and range) are provided for continuous variables, and percentages for categorical variables. To prevent assumptions on the distribution of data, the nonparametric Wilcoxon signed rank test was used to evaluate the difference in mean for continuous variables, and the *Z* test was used for comparisons of categorical variables. Logistic regression evaluating the relationship between the presence of low muscle mass and selected exploratory variables is expressed in odds ratios (OR) with 95% confidence intervals (CI). *P* values for comparisons between the groups with and without low muscle mass were calculated; *P* < 0.05 was considered statistically significant.

## Results

**Fig. 1:**
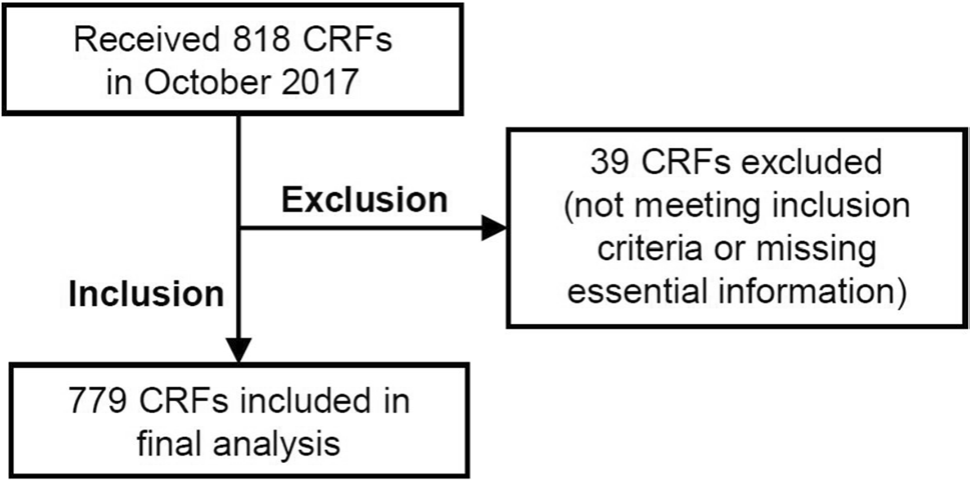
Flow diagram representing the sample population enrolled in the SCAN study. Of 818 CRFs received in October 2017, 52 were excluded as they did not meet selection criteria or lacked essential information; 766 CRFs were included in the study analysis

A participant flow diagram for this study is presented in Fig. 1.

### Low muscle mass prevalence in cancer patients and their clinical characteristics

The patients’ demographic and disease characteristics are presented in Table 1. Males and females were equally represented in the study, with an average age of 65.0 ± 11.8 years; cancer types were colon (37.1%), lung (25.5%), breast (22.6%), kidney (7.8%), and prostate (7.0%). Average times since diagnosis of the primary tumor were 46.9 ± 60.7 months. The majority of patients (40.5%) were undergoing their first line of treatment.

**Table 1:** Demographic and cancer characteristics of patients with and without low skeletal muscle mass, as assessed by the L3 computed tomography imaging method. A patient has low skeletal muscle mass if the skeletal mass index (SMI) is < 55cm^2^/m^2^ in men and < 39 cm^2^/m^2^ in women. In the total population of 766 patients, 529 were found to have low skeletal muscle mass, denoting a prevalence of 69.1%. Results are presented by *N* (%) or mean ± SD. *NS* not significant, *NA* not applicable. Non-responses are not shown

	Total patients	Low muscle mass	Unimpaired muscle mass	*P*
SMI, cm^2^/m^2^	*n* = 766	*n* = 529	*n* = 237	
Mean ± SD	43.2 ± 8.7	40.6 ± 7.6	49.1 ± 8.2	*P* < *0.01*
Male, n (%)	*n* = 766	*n* = 529	*n* = 237	
	386 (49.9)	317 (59.9)	65 (27.4)	*P* < *0.01*
Age (years)	*n* = 762	*n* = 527	*n* = 235	
Mean ± SD	65.0 ± 11.8	66.1 ± 11.8	62.5 ± 11.3	*P* < *0.01*
Site of primary tumor, n (%)	*n* = 766	*n* = 529	*n* = 237	
Colon	284 (37.1)	200 (37.8)	84 (35.4)	NS
Lung	195 (25.5)	138 (26.1)	57 (24.1)	NS
Breast	173 (22.6)	94 (17.8)	79 (33.3)	*P* < *0.01*
Kidney	60 (7.8)	46 (8.7)	14 (5.9)	NS
Prostate	54 (7.0)	51 (9.6)	3 (1.3)	*P* < *0.01*
Time since diagnosis of primary cancer (months)	*n* = 762	*n* = 529	*n* = 237	
Median (range)	23.2 (0–400.0)	20.8 (0–396.0)	28.1 (1.0–400.0)	NS
Time since diagnosis of metastatic disease (months)	*n* = 756	*n* = 524	*n* = 232	
Median (range)	14.5 (0–285.0)	13.6 (0–285.0)	17.5 (0–160.0)	NS
Main metastatic sites, n (%)	*n* = 766	*n* = 529	*n* = 237	
Hepatic	323 (42.2)	224 (42.3)	99 (41.8)	NS
Pulmonary	310 (40.5)	208 (39.3)	102 (43.0)	NS
Lymph nodes	275 (35.9)	198 (37.4)	77 (32.5)	NS
Bones	259 (33.8)	178 (33.6)	81 (34.2)	NS
Peritoneal	90 (11.7)	64 (12.1)	26 (11.0)	NS
Cerebral	81 (10.6)	67 (12.7)	14 (5.9)	*P* < *0.01*
Treatment line,* n* (%)	*n* = 766	*n* = 529	*n* = 237	
First line of treatment	310 (40.5)	207 (39.1)	103 (43.5)	NS
Second line of treatment	217 (28.3)	154 (29.1)	63 (26.6)	NS
Third or higher	208 (27.2)	144 (27.2)	64 (27.0)	NS
Non-responses	31 (4.0)	24 (4.5)	7 (3.0)	NS
Ongoing therapies, n (%)	*n* = 766	*n* = 529	*n* = 237	
Chemotherapy	530 (69.2)	375 (70.9)	155 (65.4)	NS
Hormonotherapy	72 (9.4)	48 (9.1)	24 (10.1)	NS
Targeted therapy	305 (39.8)	192 (36.3)	113 (47.7)	*P* < *0.01*
Immunotherapy	118 (15.4)	89 (16.8)	29 (12.2)	NS
Time since initiation of ongoing therapy (months)				
Chemotherapy	*n* = 516	*n* = 366	*n* = 150	
Median (range)	8.0 (0–73.0)	8.0 (0–73.0)	7.8 (0–50.0)	NS
Hormonotherapy	*n* = 68	*n* = 45	*n* = 23	
Median (range)	15.8 (0–92.0)	18.4 (0–92.0)	6.0 (0–24.0)	*P* < *0.05*
Targeted therapy	*n* = 293	*n* = 187	*n* = 106	
Median (range)	12.9 (0–73.0)	13.4 (0–73.0)	12.0 (0–57.0)	NS
Immunotherapy	*n* = 117	*n* = 88	*n* = 29	
Median (range)	6.7 (0–36.0)	6.8 (0–36.0)	6.7 (0–23.0)	NS
PS score, n (%)	*n* = 766	*n* = 529	*n* = 237	
0	252 (32.9)	157 (29.7)	95 (40.1)	*P* < *0.01*
1	353 (46.1)	250 (47.3)	103 (43.5)	NS
2	116 (15.1)	91 (17.2)	25 (10.5)	*P* = *0.02*
3	27 (3.5)	19 (3.6)	8 (3.4)	NS
Non-responses	18 (2.3)	12 (2.3)	6 (2.5)	NS

CT-defined low skeletal muscle mass was considered to be present for patients whose SMI was < 55cm^2^/m^2^ in males and < 39 cm^2^/m^2^ in females [3]. In the total population of 766 patients, 529 were assessed as to have low muscle mass, denoting a CT-defined low skeletal muscle mass prevalence of 69.1%.

Low muscle mass was more prevalent in males than females: 83.0% (317/382) versus 55.2% (212/384) (*P* < 0.01); and patients with low muscle mass were older than those without. Low muscle mass was most prevalent in prostate cancer patients (94.4%, 51/54), compared to only 54.3% (94/173) in breast cancer patients; prevalence in the kidney, colon, and lung cancer sub-groups was comparable to that of the total population (76.7%, 70.4%, and 70.8%, respectively). Patients with low muscle mass were twice more likely to have brain metastasis than their counterparts (12.7% versus 5.9%, *P* < 0.01). PS scores were poorer for patients with low muscle mass: 29.7% had a “good” PS score versus 40.1% without low muscle mass (PS 0, *P* < 0.01); 17.2% of low muscle mass patients had a “poor” PS score of 2, compared to 10.5% of patients without low muscle mass (*P* = 0.02).

No significant difference was observed in the distribution of patients with and without low skeletal muscle mass by line of treatment and in the median time since primary cancer diagnosis. A majority of patients were receiving chemotherapy at the time of the study (69.2%), followed by targeted therapy (39.8%), immunotherapy (15.4%), and hormonotherapy (9.4%).

### Malnutrition and cachexia prevalence among cancer patients with low muscle mass

**Fig. 2:**
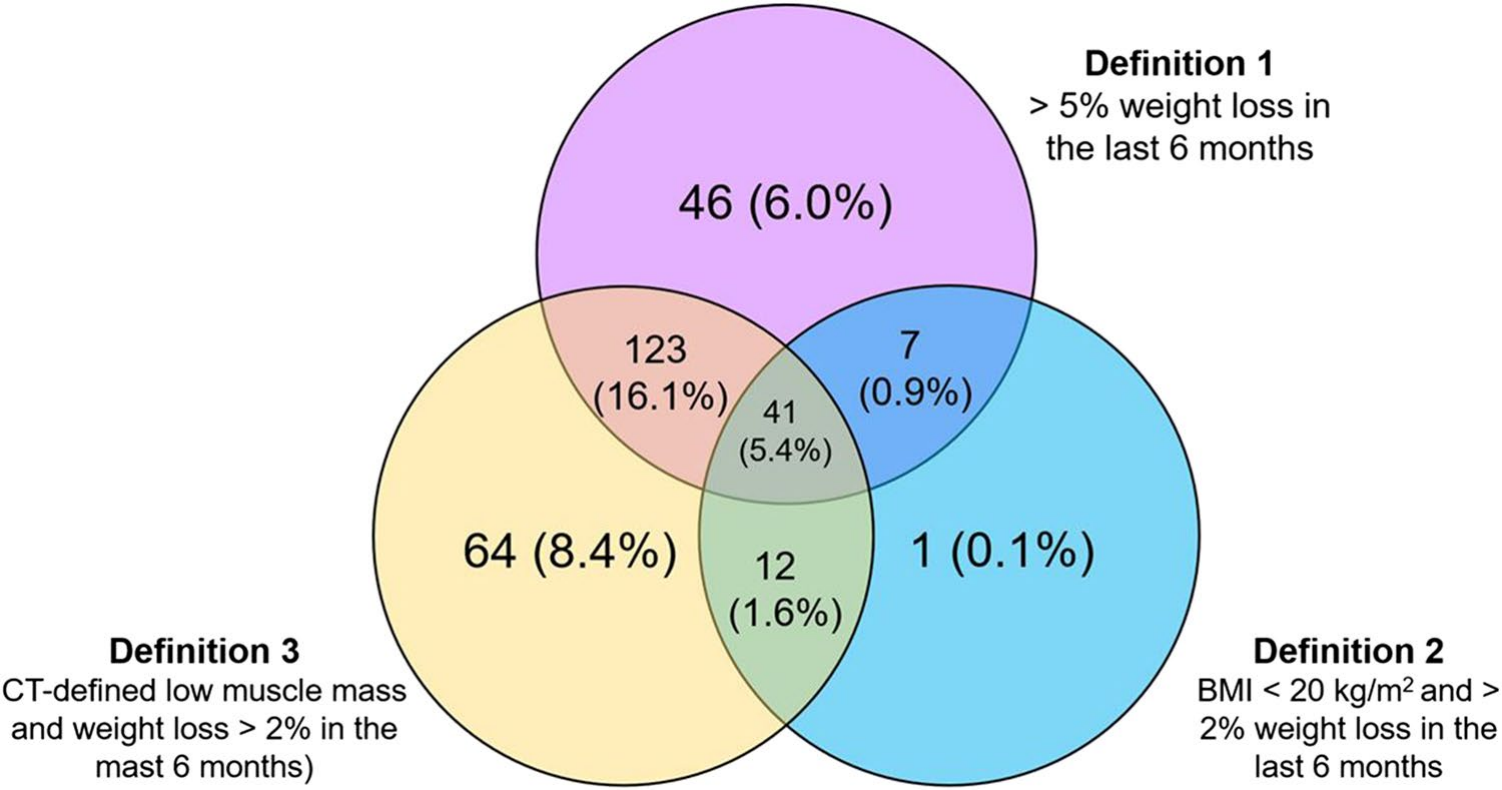
Number and proportion of cachectic patients as per the three definitions of cachexia in patients with low skeletal muscle mass (*n* = 529)

Nutrition-related data are summarized in Table 2. About four times the proportion of patients without low muscle mass were obese compared to the low muscle mass group. Food intake during the previous meal, evaluated with a VAS, revealed that 9.5% of low muscle mass versus 5.1% of unimpaired muscle mass patients had very abnormal food intake (scores 0–4, *P* = 0.04) and 9.8% of low muscle mass patients versus 2.5% of their counterparts were moderately malnourished (*P* < 0.01). Serum albumin and GPS were found to be similar between both groups; nevertheless, CRP levels were significantly elevated in low muscle mass patients (mean 29.7 ± 48.8 mg/mL versus 17.9 ± 31.3 mg/mL in patients with unimpaired muscle mass, *P* < 0.01). Cachexia prevalence in the sample was estimated at 38.4% (294/766 patients) for all three definitions by Fearon et al. [3]. Figure 2 represents the Venn diagram of the distribution of cachectic patients according to the definition to which they correspond.

**Table 2:** Nutritional status, body weight measures, and Glasgow prognostic score (GPS in metastatic cancer patients with and without low skeletal muscle mass. Results are presented as *N* (%) or mean ± SD. *NS* not significant

	Total patients	Low muscle mass	Unimpaired muscle mass	*P*
	*n* = 722	*n* = 500	*n* = 222	
Weight before cancer diagnosis (kg)—mean ± SD	73.7 ± 16.3	73.4 ± 15.4	74.2 ± 18.2	NS
	*n* = 760	*n* = 525	*n* = 235	
Current weight (kg)	69.2 ± 15.4	68.1 ± 14.5	71.6 ± 17.1	*P* < *0.01*
	*n* = 766	*n* = 529	*n* = 237	
> 2% weight loss in 1 month	157 (20.5)	120 (22.7)	37 (15.6)	*P* = *0.02*
> 5% weight loss in 1 month	59 (7.7)	46 (8.7)	13 (5.5)	NS
> 5% weight loss in 6 months	217 (28.3)	164 (31.0)	53 (22.4)	*P* = *0.01*
> 10% weight loss in 6 months	93 (12.1)	74 (14.0)	19 (8.0)	*P* = *0.03*
Current BMI (kg/m^2^) ^a^	*n* = 766	*n* = 529	*n* = 237	
Severely malnourished	20 (2.6)	17 (3.2)	3 (1.3)	NS
Moderately malnourished	58 (7.6)	52 (9.8)	6 (2.5)	*P* < *0.01*
Non-responses	10 (1.3)	6 (1.1)	4 (1.6)	NS
Obese (BMI ≥ 30 kg/m^2^)	96 (12.5)	35 (6.6)	61 (25.7)	*P* < *0.01*
	*n* = 760	*n* = 525	*n* = 235	
Mean ± SD	24.6 ± 4.6	23.7 ± 4.1	26.6 ± 5.0	*P* < *0.01*
VAS for food intake at latest meal (by the patient) ^b^	*n* = 766	*n* = 529	*n* = 237	
0–4 very poor	62 (8.1)	50 (9.5)	12 (5.1)	*P* = *0.04*
5–7 poor	199 (26.0)	146 (27.6)	53 (22.64)	NS
8–10 normal	481 (62.8)	316 (59.7)	165 (69.6)	*P* < *0.01*
Serum albumin (SA, g/l) ^a^	*n* = 766	*n* = 529	*n* = 237	
Normal	476 (62.1)	329 (62.2)	147 (62.0)	NS
Moderate malnutrition	59 (7.7)	45 (8.5)	14 (5.9)	NS
Severe malnutrition	26 (3.4)	19 (3.6)	7 (3.0)	NS
Non-responses	205 (26.8)	136 (25.7)	69 (29.1)	NS
	*n* = 564	*n* = 395	*n* = 169	
Mean ± SD	37.2 ± 6.0	37.2 ± 5.9	37.0 ± 6.2	NS
C-reactive protein (mg/l)	*n* = 381	*n* = 273	*n* = 111	
Median (range)	7.3 (0–374.0)	7.9 (0–374.0)	6.3 (0–177.0)	*NS*
Glasgow prognostic score (GPS)	*n* = 348	*n* = 249	*n* = 99	
0	166 (47.7)	113 (45.4)	53 (53.5)	NS
1	103 (29.6)	75 (30.1)	28 (28.3)	NS
2	79 (22.7)	61 (24.5)	18 (18.2)	NS

^**a**^Nutritional status based on patient BMI and serum albumin (SA) was calculated according to the following thresholds^14^:

**Moderate malnutrition**

- < 70 years old: BMI ≤ 18.5 to 16 kg/m^2^ or SA < 30 to 20 g/L

- ≥ 70 years old: BMI < 21 to 18 kg/m^2^ or SA < 35 to 30 g/L

**Severe malnutrition**

- < 70 years old: BMI ≤ 16 kg/m^2^ or SA < 20 g/L

- ≥ 70 years old: BMI < 18 kg/m^2^ or SA < 30 g/L

^**b**^VAS: 0 (no food intake)–10 (normal food intake)

### Multivariate analysis of the determinants of low muscle mass in cancer patients

Only patients for whom serum albumin data were available (*n* = 561) were included in the multivariate analysis, as this was an exploratory variable (see the “Methods” section). Associations were found between low muscle mass and moderately malnourished status (OR 5.28, 95%CI 1.74–16.00, *P* < 0.01), severely malnourished status (OR 5.00, 95%CI 1.05–23.82, *P* = 0.04), and age ≥ 70 years (OR 2.13, 1.27–3.56, *P* < 0.01). Females (OR 0.23, 95%CI 0.14–0.38, *P* < 0.01) and those not having a brain metastatic site (OR 0.26, 0.11–0.58, *P* < 0.01) were found to have a lower risk of low muscle mass (Table S1).

### Low muscle mass diagnosis and management in real-life practice

**Fig. 3:**
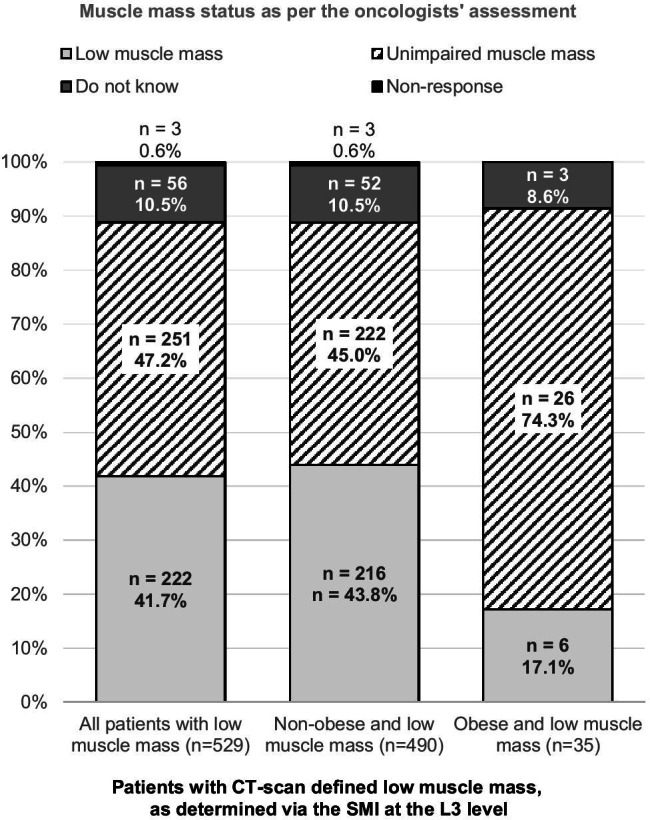
Skeletal muscle mass status as per the oncologists’ assessment. Oncologists’ diagnosis of low skeletal muscle mass is superposed to the L3 scan method using a stacked bar graph for the total low muscle mass population and for low muscle mass non-obese and obese sub-populations. Concordant and non-concordant oncologist assessments were calculated as a percentage of the low muscle mass population (y-axis); 249 of 529 patients with low skeletal muscle mass (47.1%) were incorrectly evaluated as having low muscle mass by their oncologist; 220 of 490 (44.9%) of non-obese low muscle mass patients were incorrectly evaluated. Among obese patients, the discordance was much higher, with 26 out of 35 (74.3%) of low muscle mass patients being erroneously assessed as not having low skeletal muscle mass

Figure 3 presents the level of disagreement between low muscle mass diagnosis via SMI measurement and the oncologist’s assessment of low muscle mass. Whereas underestimation by physicians in non-obese patients with low muscle mass (44.9%) was similar to that found in the total population, non-diagnosis in obese cancer patients was much higher, reaching 74.3% (*P* < 0.01). Over a third of patients with low muscle mass were receiving personalized dietetic counselling compared to 24.1% of patients with unimpaired muscle mass (*P* < 0.01). Furthermore, a higher proportion of patients with low muscle mass (15.9%) had future nutritional counselling planned by their oncologist, compared to those with unimpaired muscle mass (6.1%, *P* < 0.01). Nutritional management was available to 28.4% of patients with low skeletal muscle mass versus those without (13.9%, *P* < 0.01); 15.9% of all patients in the study were availing from physiotherapy, with no significant differences between patients with and without low muscle mass. Mean patient-reported daily spent-in-bed time was also higher for low muscle mass patients (10.3 ± 3.3 h/day) than those without low muscle mass (9.3 ± 2.9 h/day, *P* < 0.01) (Table 3).

**Table 3:** Nutritional management and physical activity interventions in metastatic cancer patients, with and without low skeletal muscle mass. Unknown responses and non-responses are not reported in the table

	Total patients	Low muscle mass	Unimpaired muscle mass	*P*
Patients currently receiving personalized nutrition counselling from a nutritionist, dietician or healthcare professional	*n* = 766	*n* = 529	*n* = 237	
Yes	237 (30.9)	180 (34.0)	57 (24.1)	*P* < *0.01*
No	527 (68.8)	347 (65.6)	180 (75.9)	
If no, is any personalized nutritional counselling planned?	*n* = 527	*n* = 347	*n* = 180	
Yes	66 (12.5)	55 (15.9)	11 (6.1)	*P* < *0.01*
Patients currently under specific nutritional management	*n* = 766	*n* = 529	*n* = 237	
Yes	183 (23.9)	150 (28.4)	33 (13.9)	*P* < *0.01*
Oral nutritional supplements	172 (22.5)	140 (26.5)	32 (13.5)	*p* < *0.01*
Enteral nutrition	6 (0.8)	6 (1.1)	0 (0.0)	NS
Parenteral nutrition	7 (0.9)	6 (1.1)	1 (0.4)	NS
No	580 (75.7)	377 (71.3)	203 (85.7)	*P* < *0.01*
If NO, is any personalized nutritional management planned?	*n* = 580	*n* = 377	*n* = 203	
Yes	56 (9.7)	44 (11.7)	12 (5.9)	*P* = *0.05*
Current patient physical activity ^a^				
Number of hours spent in bed per day (hours/day) ^a^	*n* = 591	*n* = 412	*n* = 179	
Mean ± SD	10.0 ± 3.2	10.3 ± 3.3	9.3 ± 2.9	*P* < *0.01*
≥ 11 h bedridden per day	185 (31.2)	141 (34.2)	44 (24.6)	*P* < *0.05*
Patients practicing physical exercises	*n* = 628	*n* = 429	*n* = 199	
Frequency of 30-min physical exercises per week^b^ [median (range)]	1.2 (0–35)	1.1 (0–28)	1.4 (0–35)	NS
	*n* = 766	*n* = 529	*n* = 237	
Patients undertaking physiotherapy	122 (15.9)	86 (16.3)	36 (15.2)	NS
Patients consulting a sports coach	22 (2.9)	12 (2.3)	10 (4.2)	NS

^**a**^Including sleeping time

^b^Equivalent to 30 min of walking or cycling on flat terrain, or swimming

### Impact of low muscle mass on ongoing cancer therapy tolerability

Dose reductions, treatment interruptions, and occurrence of AE of grade 3 and greater related to ongoing treatment were comparable between low muscle mass patients and their counterparts. However, a higher number of low muscle mass patients (11.9%) experienced treatment delays due to toxicities, compared to patients without low muscle mass (6.8%, *P* = 0.04). Low muscle mass patients were also significantly more likely to have experienced at least two treatment-related AEs (13.8% versus 8.4% among their counterparts; *P* = 0.03) (Table S2).

## Discussion

Our prospective study represents the largest multicenter sample of metastatic cancer patients collected in France, assessing low muscle mass prevalence among 766 patients. As previously reported with different cut-offs for SMI [24, 25], low muscle mass was found to be highly prevalent among these cancer patients (69.1%). These data demonstrate how commonly available CT imagery may be used to objectively measure muscle loss, thus facilitating diagnosis and subsequent treatment of this common condition in cancer patients.

We found that low muscle mass was significantly more prevalent in males than in females. Multivariate analyses also confirmed a strong association between low muscle mass prevalence and sex, with females being at lower risk. A similar sex-based association has been previously reported in other cancer sarcopenia populations [26–28]. In this study, low muscle mass prevalence was found to be highest in prostate cancer (94.4%) and lower in breast cancer patients (54.3%). Indeed, prostate cancer patients have multiple risk factors for muscle mass loss, such as advanced age, advanced cancer, and testosterone deprivation. In our study, prostate cancer patients had a mean age of 70.6 ± 8.5 versus 60.4 ± 13.4 years in breast cancer patients. Among sex-neutral cancers in this study, low skeletal muscle mass was similarly prevalent in each sex. Multivariate analysis did not reveal any associations between cancer type and the presence of low muscle mass.

The choice of the cut-off may be another factor explaining the differences in low muscle mass prevalence by sex. In studies where notably different SMI cut-off values were used, no significant differences in low muscle mass prevalence by sex was identified, such as in a recent study by Martin et al. [29], where L3 SMI thresholds for low muscular mass were stratified by BMI and sex, as can be seen when comparing the cut-off value that was employed in our work with those from 2 other studies based in Western populations [25, 26]. In all cases, a very high prevalence of low muscle mass was found in this French population (> 58%; Table S3).

A 2016 literature review reported a prevalence of low muscle mass ranging from 5 to 89%, depending on the malignancy and methods used for low muscle mass diagnosis [30]. Elsewhere, a prevalence of 25–30% has been reported, equivalent to about 1 million cancer patients in Europe [31]. There could also be other confounding factors influencing the apparent differences in low muscle mass prevalence by sex, such as biological and behavioral characteristics (hormones, immune responses, smoking, alcoholism, food habits). Nevertheless, an analysis that accounts for confounding factors was beyond the scope of our study, which is not longitudinal and was intended to be descriptive.

Our study is the first to demonstrate a potential association between brain metastases and low muscle mass. Patients with cerebral metastases are often prescribed corticosteroids, resulting in greater fatigue, and they also are at risk of developing hemiplegia. Both factors may lead to an overall reduction in the patients’ physical activity, increasing the risk of low muscle mass.

Over 8 out of 10 cachectic patients had CT-defined low muscle mass (240 of 294 cachectic patients). This finding thus vouches for the use of CT imagery to diagnose low muscle mass, thereby promising a more reliable detection of cachexia.

The most worrying result of our study is that despite its high prevalence, practitioners were unable to recognize low skeletal muscle mass in nearly half of those patients with low muscle mass during the survey, even while completing nutritional status data. Low muscle mass was more poorly recognized in obese patients (Fig. 3). Our findings also demonstrate the general lack of nutritional and physical therapy support available to cancer patients in France (Table 3), as not more than a third of patients with low muscle mass were receiving nutritional counselling or nutritional management (in the form of supplements, enteral or parenteral nutrition). Less than 1 out of 5 patients with low muscle mass were undergoing physiotherapy. Low muscle mass patients have a higher risk of being bedridden for longer durations and having abnormal food intake. It is well known that suitable nutritional counselling and management and physical exercise regimes are helpful countermeasures to prevent muscle wasting, and hence cachexia development [1, 32].

These observations on nutritional support are sadly not new, as highlighted in the cross-sectional NutriCancer2012 study [33, 34] that evaluated malnutrition prevalence in over 2000 cancer patients in France. Malnutrition prevalence was around 40%, and it was often diagnosed belatedly. Approximately 10% of these patients lacked any type of nutritional management, and there was only 70% concordance between the patients’ true conditions and the physicians’ evaluations. The study explained that such a low recourse to nutritional management was due to a lack of knowledge on malnutrition diagnosis and nutritional treatments (64% of interviewed physicians) and the lack of a nutrition team in the hospital (56% of physicians) [35]. While we could describe the existing nutrition and physical activity trends in patients with and without low muscle mass as part of this study, a longitudinal study would be better suited to investigate the link between these factors, their development and progression.

Low muscle mass patients have been reported to be more likely to accumulate treatment-related events [36–38]. In our study, however, no links could be established with dose reductions, treatment interruptions, and occurrence of grade 3 AEs. We did find an effect of low muscle mass on increased delays in treatment administration due to treatment-related toxicities. We suggest that our study design was such that it favored the selection of patients with low toxicity prevalence. This might also be explained by the relative heterogeneity in our sample with regard to ongoing treatment and cancer type (compared to other studies assessing low muscle mass and treatment toxicity [36–38]). However, the principal objective of this study was not to assess such toxicity, but to evaluate low muscle mass prevalence in a population of patients with metastatic cancers most frequently encountered in clinical settings.

We aimed to demonstrate the prevalence and impact of low muscle mass in a population of metastatic cancer patients in various healthcare settings in France. Measures taken to ensure data quality included the study’s multi-centric design, large sample size, oncologist and radiologist training in data collection, and collaboration with a coordinating center. However, some limitations were duly noted. This was a cross-sectional study that evaluated a patient’s low muscle mass status at a certain timepoint, using their medical records to obtain much of the historical data reported. Such a study design offers limited control over center-based variability in the level of completeness and methods of measuring and recording of past data. Additionally, a cross-sectional design is inadequate for obtaining data on the development and evolution of cancer-related low muscle mass over the course of a person’s illness, the evaluation of contributing factors, and its eventual impact on patient outcomes. Future longitudinal prospective studies, with reasonable follow-up durations, will be required to better understand this complex disorder.

It is possible that using cut-offs defined in a Canadian population could lead to an overestimated prevalence of low muscle mass in French patients, due to differences in population body weight demographics. Therefore, the use of the Canadian thresholds to evaluate the prevalence of muscle mass loss is one of the limitations of our study. Although the thresholds referred to in this study have been widely accepted in the literature, they are based on North American populations. This could lead to the risk of over- or under-estimations of prevalence when applied to our European study population. It has been established, for example, that cut-offs used for Western populations differ from those used for East Asian populations due to different morphotypes [39]. France and Canada, however, both represent Western populations and Martins et al. [14] have weighted their calculations using BMI (Table S3) to help account for different rates of obesity between these populations. A histogram depicting the SMI distribution among males and females in the SCAN study (Figure S1) shows that the mean SMI for females is 39.7 cm^2^/m^2^ and the mean SMI for males is 47.1 cm^2^/m^2^.

Since this study was performed across 29 centers, where different software was used to determine SMI, the inter-center precision of CT readings must be addressed. Recent studies have shown that CSA measurements may vary along with CT acquisition parameters; nevertheless, these variabilities would be clinically insignificant for the assessment low muscle mass [40, 41]. Additionally, the decision to allow the use of different post-treatment stations (involving the extraction of measurements within the established threshold of − 29 to + 150 HU followed by manual segmentation) was supported by data from a study that compared four dedicated post-treatment stations and demonstrated an excellent correlation between readings, with very limited inter- and intra-operator variability [18]. Moreover, the effect of contrast media and varying slice thickness on differences in body composition assessments had to be considered [16, 42], which is why mandatory distance training was provided to the investigating radiologists to standardize these measurements as much as possible. If the objective of the study had been a longitudinal monitoring of muscle mass changes, the type of CT parameters, post-treatment stations, contrast media, and slice thickness choices would have been standardized across centers. However, for a cross-sectional study seeking to describe the real-life prevalence of low skeletal muscle mass, these limitations were considered acceptable for the purpose of the investigation.

Another methodological limitation is the lack of standardization (and possibly even homogeneity) of the oncologists’ chosen methods of low muscle mass diagnosis, which limited our ability to compare the accuracy of their diagnosis relative to CT-defined low muscle mass. Since there is no standard method of evaluating low muscle mass in cancer patients in France, oncologists were permitted to assess this as per their knowledge and experience. The results of this descriptive analysis should therefore be interpreted with caution.

## Conclusions

Low muscle mass was found to be highly prevalent, at 69.1%, in patients with metastatic cancers in French clinical practice as evaluated by L3 level CT of muscle mass measurements. Nutritional and physical therapy support were only provided to a minority of patients with low skeletal muscle mass. Low muscle mass was often underdiagnosed by the oncologist, even more so in obese patients, highlighting a worrying gap in the oncologists’ understanding of muscle wasting diagnosis and management. Our findings underline the necessity for improvements in oncologist education of diagnostic tools and the nutritional aspects of cancer management, and the need for an increased involvement of radiologists, dieticians, nutritionists, physiotherapists, and fitness counsellors in the regular, long-term management of cancer-related low muscle mass as part of a more wholistic standard of care.

## Supplementary Information

ESM 1 (DOCX 45 kb)

## Data Availability

N/A.

## Code availability

N/A.
