# Supplementary material for: The prevalence of CT-defined low skeletal muscle mass in patients with metastatic cancer: a cross-sectional multicenter French study (the SCAN study)
Source: Support Care Cancer. 2021 Dec 3;30(4):3119–29. doi: 10.1007/s00520-021-06603-0 (PMC8857123; doi:10.1007/s00520-021-06603-0)
Supplement: Supplementary file 1 — (DOCX 45 kb) [file 520_2021_6603_MOESM1_ESM.docx]

The prevalence of CT-defined low skeletal muscle mass in patients with metastatic cancer: a cross-sectional multicenter French study (the SCAN Study)

***Supportive Care in Cancer***

**Authors:**

Bruno Raynard, Frederic Pigneur, Mario Di Palma, E. Deluche and François Goldwasser.

**Corresponding author:**

Bruno Raynard.

Address: Gustave-Roussy, 114 Rue Edouard Vaillant, 94800 Villejuif, France

Telephone: +33 (0)1 42 11 56 12

Email: [Bruno.raynard@gustaveroussy.fr](mailto:Bruno.raynard@gustaveroussy.fr)

**Supporting Information**

**Table S1** Explanatory variables for the multivariate analysis of factors associated with the presence of CT-defined low muscle mass in metastatic cancer patients

**A:** Categorical variables that were selected for multivariate analysis and their conditions

| ***Variable*** | ***Categories*** |
| --- | --- |
| Age ^a^ | 18 – 59 / 60 – 64 / 65 – 69 / > 70 years |
| Sex | Male or Female |
| Tumour location | Lung / Kidney / Colon / Breast / Prostate |
| Presence of cerebral metastasis | Yes or No |
| Weight loss after 1 month of diagnosis ^a^ | > 5% or ≤ 5% |
| Weight loss after 6 months of diagnosis ^a^ | > 10% or ≤ 10% |
| Current BMI ^a, b^ | Normal BMI / Moderate malnutrition / Severe malnutrition |
| Serum albumin (SA) ^a, c^ | Normal / Moderate malnutrition / Severe malnutrition |
| PS score | 0 / 1 / 2 / 3 / 4 |
| VAS for food intake at last meal ^a^ | 0 – 4 / 5 – 7 / 8 – 10 |

**^a^ Continuous or discrete variable that were coded into categorical variables for multivariate analysis**

**^b^ Malnutrition categories for BMI ^14^**

**Moderate malnutrition:** < 70 years old: BMI ≤ 18.5 to 16 kg/m^2^ **;** ≥ 70 years old: BMI < 21 to 18 kg/m^2^

**Severe malnutrition:** < 70 years old: BMI ≤ 16 kg/m^2^ ; ≥ 70 years old: BMI < 18 kg/m^2^

**^c^ Malnutrition categories for serum albumin ^14^**

**Moderate malnutrition:** < 70 years old: SA < 30 to 20 g/L **;** ≥ 70 years old: SA < 35 to 30 g/L

**Severe malnutrition:** < 70 years old: SA < 20 g/L **;** ≥ 70 years old: SA < 30 g/L

**B:** Factors found to be associated with the presence of CT-defined low muscle mass in metastatic cancer patients after multivariate analysis

| ***Variable*** | ***Modalities*** | ***Probability (p)*** | ***Odds ratio (OR)*** | ***95% CI*** |
| --- | --- | --- | --- | --- |
| Age | 60 to 64 years old | 0.02 | 2.09 | 1.14 – 3.83 |
| Age | 70 years old and more | < 0.01 | 2.13 | 1.27 – 3.56 |
| Current BMI | Moderate malnutrition | < 0.01 | 5.28 | 1.74 – 16.00 |
| Current BMI | Severe malnutrition | 0.04 | 5.00 | 1.05 – 23.82 |
| Tumor localization | Prostate | 0.02 | 10.43 | 1.36-80.32 |
| Gender | Female | < 0.01 | 0.23 | 0.14 – 0.38 |
| Metastatic sites | No cerebral metastasis | < 0.01 | 0.26 | 0.11 – 0.58 |

**Table S2** Impact of CT-defined low muscle mass on ongoing anti-cancer treatment related toxicities and treatment management. *p* values are given for comparisons between the low muscle mass and unimpaired muscle mass groups. The proportion of patients who underwent treatment modifications or adverse events (AE) due to treatment toxicities are presented below by number of each event experienced for 4 types of events: 1) dose reduction, 2) treatment interruptions, 3) delayed treatment due to toxicity and 4) an AE ≥ grade 3. The only event type for which low muscle mass status was found to have a statistically significant impact on occurrence in cancer patients was treatment delays due to toxicity (*p* = 0.04).

|  | ***Total***  ***n = 766*** | ***Low muscle mass patients***  ***n = 529*** | ***Unimpaired muscle mass patients***  ***n = 237*** | ***p*** |
| --- | --- | --- | --- | --- |
| 0 events linked to treatment | *536 (70.0)* | *362 (68.4)* | *174 (73.4)* | *NS* |
| 1 event linked to treatment | *114 (14.9)* | *78 (14.7)* | *36 (15.2)* | *NS* |
| 2 events linked to treatment | *56 (7.3)* | *45 (8.5)* | *11 (4.6)* | *NS* |
| 3 events linked to treatment | *24 (3.1)* | *18 (3.4)* | *6 (2.5)* | *NS* |
| All 4 events linked to treatment | *13 (1.7)* | *10 (1.9)* | *3 (1.3)* | *NS* |
| ≥ 2 events linked to treatment | 93 (12.2) | 73 (13.8) | 20 (8.4) | *P*=0.03 |
| Non-responses | *23 (3.0)* | *16 (3.0)* | *7 (2.9)* | *NS* |
| **Patients with treatment delay due to toxicities during the previous month** | | | | |
| Yes | *79 (10.3)* | *63 (11.9)* | *16 (6.8)* | *P=0.04* |

Figure S1. Skeletal muscle index distribution (SMI) in men (n=386) and women (n=387)


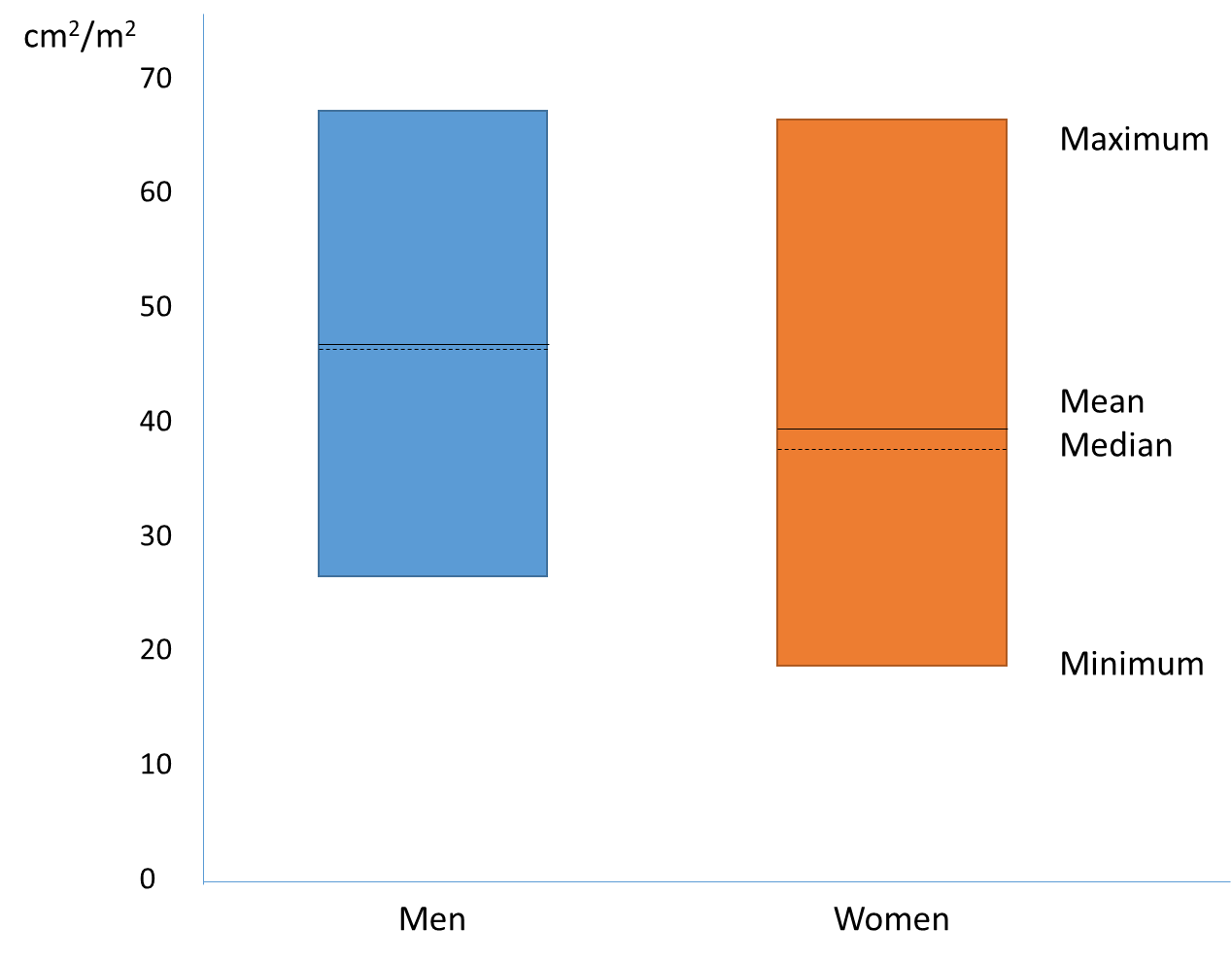


**Table S3: Impact of the L3 SMI cut-off value on the profile of metastatic cancer patients**

Patient characteristics are presented for males and females as per each of the three L3 SMI cut-offs.

|  | **Cut-off 1 ^a^** | | **Cut-off 2 ^b^** | | **Cut-off 3 ^c^** | |
| --- | --- | --- | --- | --- | --- | --- |
| **Low Muscle Mass**  **prevalence** | **69.1%** | | **62.5%** | | **58.6%** | |
|  | **Low**  **Muscle Mass**  **(n = 529)** | **Normal**  **Muscle Mass**  **(n = 237)** | **Low**  **Muscle Mass**  **(n = 479)** | **Normal**  **Muscle Mass**  **(n = 287)** | **Low**  **Muscle Mass**  **(n = 449)** | **Normal**  **Muscle Mass**  **(n = 314)** |
| **Male** | 59.9% * | 27.4% * | 58.5% * | 35.5% * | 45.2% * | 56.1% * |
| **Female** | 40.1% * | 72.6% * | 41.5% * | 64.5% * | 54.8% * | 43.9% * |
| **Age, Mean ± SD** | 66.1 ± 11.8 * | 62.5 ± 11.3 * | 66.2 ± 11.8 * | 63.0 ± 11.5 * | 66.0 ± 12.1 * | 63.5 ± 11.2 * |
| Duration since cancer diagnosis, Mean ± SD | 45.7 ± 61.4 | 49.7 ± 58.9 | 47.5 ± 63.5 | 46.0 ± 55.6 | 51.4 ± 67.4 * | 40.4 ± 48.8 * |
| **Tumor location** | | | | | | |
| Lung | 26.1% | 24.1% | 26.7% | 23.3% | 24.1% | 27.4% |
| Kidney | 8.7% | 5.9% | 8.8% | 6.3% | 7.8% | 7.6% |
| Colon | 37.8% | 35.4% | 36.1% | 38.7% | 35.0% | 40.4% |
| Breast | 17.8% * | 33.3% * | 18.6% * | 29.3% * | 24.3% | 20.4% |
| Prostate | 9.6% * | 1.3% * | 9.8% * | 2.4% * | 8.9% * | 4.1% * |
| **PS Score** | | | | | | |
| 0 | 29.7% * | 40.1% * | 28.6% * | 40.1% * | 30.1% * | 36.9% * |
| 1 | 47.3% | 43.5% | 47.8% | 43.2% | 46.1% | 46.3% |
| 2 | 17.2% * | 10.5% * | 17.3% * | 11.5% * | 18.0% * | 11.1% * |
| 3 | 3.6% | 3.4% | 3.8% | 3.1% | 4.0% | 2.9% |

**^a^ Cut-off 1 (SCAN study):** Low muscle mass if total muscle SMI at L3 is < 55 cm²/m² in males and < 39 cm²/m² in females [3].

**^b^ Cut-off 2:** Low muscle mass if total muscle SMI at L3 is < 52.4 cm²/m² in males and < 38.5 cm²/m² in females [25].

**^c^ Cut-off 3**: Low muscle mass if total muscle SMI at L3 is < 43 cm²/m² in males with BMI < 25.0 OR < 53 cm²/m² in males with BMI ≥ 25. In females: SMI < 41 cm²/m² is considered low muscle mass [26].

**^*^** *P* < 0.01 for the difference observed between Low muscle mass and Normal muscle mass patients
